# A neural network-based PDE solving algorithm with high precision

**DOI:** 10.1038/s41598-023-31236-0

**Published:** 2023-03-18

**Authors:** Zichao Jiang, Junyang Jiang, Qinghe Yao, Gengchao Yang

**Affiliations:** School of Aeronautics and Astronautics, Sun Yat-sen University, Guangzhou, 510275 China

**Keywords:** Computational science, Applied mathematics

## Abstract

The consumption of solving large-scale linear equations is one of the most critical issues in numerical computation. An innovative method is introduced in this study to solve linear equations based on deep neural networks. To achieve a high accuracy, we employ the residual network architecture and the correction iteration inspired by the classic iteration methods. By solving the one-dimensional Burgers equation and the two-dimensional heat-conduction equation, the precision and effectiveness of the proposed method have been proven. Numerical results indicate that this DNN-based technique is capable of obtaining an error of less than 10^–7^. Moreover, its computation time is less sensitive to the problem size than that of classic iterative methods. Consequently, the proposed method possesses a significant efficiency advantage for large-scale problems.

## Introduction

The numerical methods for solving partial differential equations (PDEs) are among the most challenging and critical engineering problems. The discrete PDEs form sparse linear equations and are usually solved by iteration methods, e.g., the Gauss–Seidel method^1^, the conjugate gradient (PCG) method, etc.^2–4^. Classic iterative solvers assure the precision and the reliability of the solutions but bring the challenge in terms of computational consumption.

In recent years, deep neural network (DNN), which reveals superior capability to process and to predict complicated systems, has been widely employed in a variety of fields, e.g., natural language processing^5^, computer vision^6^, etc.^7,8^ Due to its high computational efficiency and scalability, especially on heterogeneous platforms, DNN has become a promising technique in scientific computing and even provides the possibility for real-time PDE solving^9^. Ray et al.^10^ proposed a neural network-based indicator to correct the irregular solution in the discontinuous Galerkin scheme. Chan et al. and Wang et al.^11,12^ employed neural networks to solve multiscale problems. In the numerical simulation of engineering problems, DNN is utilized as a direct solver of the approximated system, and solutions are found with high efficiency^13–16^.

The idea of utilizing DNN to achieve numerical solutions of PDEs brings certain potential risks as well. According to the universal approximation theorem, the piecewise continuous objective function is a necessary condition for the reliability of DNN approximation^17,18^. However, solutions of the PDEs, particularly the nonlinear PDEs, do not always satisfy the regularity condition^19^. Accordingly, the solutions achieved from the direct DNN prediction usually have inadequate numerical accuracy. Therefore, many published neural network-based studies focus on semi-analytic models as an alternative to the direct prediction. For instance, Raissi et al.^20^ first proposed physics-informed neural network (PINN) to solve PDEs by embedding integral forms into the loss function. Ehsan et al.^21^ developed the hp-VPINN, an improved version of PINN with a higher accuracy. Ew^22^ employed the DNN to solve variational problems that arise from PDEs. These methods translate the original PDEs into parametric models and design the penalty functions to ensure the compatibility of the DNN models with the original PDEs and improve the accuracy of the DNN-based solutions but their accuracy is usually limited. Some recent studies have employed novel models that have been employed in other areas, e.g. using ConvLSTM^23^ and data-driven GMG^24^ to solve Naiver-Stokes equation, but their lowest error is in the range of 10^–3^–10^–2^.

Solving the intermediate linear equations through DNN is another intuitive and enlightening idea to combine the advantages of neural networks and classic numerical methods. Replacing the classic iteration methods like PCG and GMRES^25^ with DNN can directly accelerate the computational process of the finite element method (FEM), the finite difference method (FDM), and the spectral methods. For instance, Xiao et al.^26^ proposed a neural network-based solver to expedite the FDM solving process of the Poisson equation in fluid simulations. Following this approach, the network's architecture and the linear equations' regularity are the essential factors, and the former will be discussed in detail later in this manuscript. The regularity of the intermediate linear equations is usually controllable and readily quantifiable relative to the original PEDs or their parametric models. Therefore, solving the intermediate linear equations by DNN has potentially better improvement in terms of precision.

Interpretability^27^ is another critical issue for the application of DNN in PDE, which can be comprehended as an explanation of how a particular input leads to a particular output in DNN and what factual information the DNN learned during training. The interpretability of a generic DNN model is still an open problem, which inevitably restricts the application of DNNs in numerical computation. However, DNN is proven sufficiently reliable as an inner interpolation when the training set is sampled uniformly enough to support almost all possible inputs. The homogeneous data set of linear equations is fortunately convenient to generate. Moreover, for a particular algorithm, the discretization scheme of the PDE is usually unique; thus, the corresponding linear equations are constrained to a small range. Therefore, the DNN-based solvers of the intermediate linear equations effectively avoid the challenge of interpretability.

Regarding the network architecture, the classic DNN model suffers from network degradation and is consequently unattainable to obtain high accuracy^28^. Specifically, the higher depth of the network does not improve the accuracy but even has a negative effect. To fix the problem of network degradation, He et al. proposed Res-Net^28^. A short connection called Res-block covering the hidden layers is involved in the network to maintain the training information transition to each hidden layer. It is proved effective in many practical applications, for example, Transformer proposed by Google^29^. In numerical computation and PDE, Tong et al. employed Res-Net in the simulations of the linear and nonlinear self-consistent systems^30^. Res-Net was also utilized by Ew^22^, mentioned above.

This paper presents an innovative DNN-based algorithm to solve linear equations with high precision and efficiency. The basic idea of this method is to solve the linear equations based on DNN, as a substitute for the classic iteration algorithms. The proposed method maintains the high efficiency of DNN and assures the necessary precision for PDE solving via the following two aspects. Firstly, we employed the Res-Net architecture in the DNN model; thus, the accuracy can improve with the increasing depth of the network. Moreover, inspired by classic residual correction methods, we combined the DNN model with a correction iteration process, which reduces the error of the results from DNN iteratively.

To balance the network architecture's simplicity and the algorithm's generality, we focus on multi-diagonal linear equations. As one of the most common types of matrices, the multi-diagonal matrix is the discrete form of various PDEs. In FDM, many schemes correspond to differential equations with a naturally multi-diagonal structure. The equations can be further reduced even to tridiagonal equations when combined with the alternating direction implicit (ADI) method. For instance, both the Burgers equation and the heat-conduction equation have a 2nd-order precision scheme satisfying this property. More importantly, several vectors can directly represent a multi-diagonal equation, implying that an intuitive network input layer can be constructed based on this feature.

The DNN model predicts the solution of the equation based on the input set of vectors representing the equation and offers another vital performance advantage. In FDM, solving several linear equations with the same structure is often necessary. For example, in the ADI method, such a process exists at each time step. Traditional methods usually require circular construction of the matrix, which brings unavoidable additional computational time overhead. The algorithm, however, can solve multiple equations simultaneously, effectively avoiding this problem.

This paper is organized as follows: the main ideas are introduced in section "Methodology". In section "Precision-oriented parametric analysis", we investigate several factors affecting the numerical precision. Section "Numerical experiments and validation" presents the numerical results that demonstrate the precision and efficiency of the proposed method. The conclusions are drawn in section "Conclusion".

## Methodology

Inputting equations in matrix form into a DNN model and improving the accuracy are two critical subroutines in the proposed algorithm. In this section we present a dimension-reduced representation of the matrices in FDM, which is also utilized as input to the DNN model. In design of the DNN model, we employ the Res-Net architecture mentioned in section "Introduction" to address some of the difficulties of DNN models, e.g. network degradation. However, the optimization of the network is not sufficient to improve the accuracy to that required for numerically solving the PDE, and we have therefore developed the iteration algorithm that will be introduced in detail in section "A correction iteration".

### The representation of the linear equation

The inputs of a DNN model are usually vectors, whereas the linear equations in FDM are always in matrix form, thus we first consider how to convert the equations into a form that can be fed into the DNN model. Since the linear equations in FDM describe the relationships between neighboring nodes, the matrices are sparse and have fixed and regular non-zero element positions, which inspired us to adopt a dimension-reduced matrix representation.

In FDM, the discrete equations generally form

1 $${\sum }_{d=1}^{D}{a}_{0}^{\left(d\right)}{x}_{{\varvec{p}}}+{\sum }_{d=1}^{D}{\sum }_{m=-k}^{k}{a}_{m}^{\left(d\right)}{x}_{{\varvec{p}}+m{{\varvec{e}}}^{\left(d\right)}}={f}_{{\varvec{p}}},$$

where $D$ is the dimension and ${\varvec{p}}\in {\mathbb{Z}}^{D}$ is the index vector of the node, ${{\varvec{e}}}^{\left(d\right)}$ is the $d$-th unit vector, ${x}_{{\varvec{p}}}$ is the solution while ${f}_{{\varvec{p}}}$ is the righthand term at node ${\varvec{p}}$. ${a}_{m}^{\left(d\right)}\left(d\le D, m\le k\right)$ represents the constant coefficients of the difference scheme, where is the order of the scheme.

In this paper, we focus on the PDEs in rectangular domains in Cartesian coordinate systems, where the difference equations are correspondingly multi-diagonal and sparse. The choice of matrix representation is particularly important for the solving process, which drives us to adopt a formulation of the multidiagonal matrixes that is more appropriate for the neural networks.

Considering an arbitrary linear system $A{\varvec{x}}={\varvec{f}}$, the left-hand matrix $A$ can be decomposed in the form of

2 $$\begin{array}{c}A={\sum }_{i=0,\pm 1,\pm 2,\cdots }{\mathrm{diag}}_{i}\left({a}_{i,1},{a}_{i,2},\cdots {a}_{i,\left(n-i\right)}\right) \\ =\left[\begin{array}{ccccc}{a}_{\mathrm{0,1}}& {a}_{\mathrm{1,1}}& \cdots & {a}_{\left(n-2\right),1}& {a}_{\left(n-1\right),1}\\ {a}_{-\mathrm{1,1}}& {a}_{\mathrm{0,2}}& {a}_{\mathrm{1,2}}& \ddots & {a}_{\left(n-2\right),2}\\ \vdots & {a}_{-\mathrm{1,2}}& {a}_{\mathrm{0,3}}& \ddots & \vdots \\ {a}_{-(n-2),1}& \ddots & \ddots & \ddots & {a}_{1,\left(n-1\right)}\\ {a}_{-(n-1),1}& {a}_{-(n-2),2}& \cdots & {a}_{-1,\left(n-1\right)}& {a}_{0,n}\end{array}\right],\end{array}$$

where the symbol ${\mathrm{diag}}_{i}\left(\cdot \right)$ denotes the matrix that places the elements of the vector $\left({a}_{i,1},{a}_{i,2},\cdots {a}_{i,\left(n-i\right)}\right)$ on the *i*-th diagonal. In particular, ${\mathrm{diag}}_{0}\left(\cdot \right)$ represents the main diagonal, ${\mathrm{diag}}_{i}\left(\cdot \right) (i>0)$ is the diagonal above the main diagonal, and oppositely ${\mathrm{diag}}_{i}\left(\cdot \right) \left(i<0\right)$ is the lower diagonal elements.

The matrix in (2) can be represented by a series of vectors, called diagonal vectors, and the number of diagonal vectors coincides with the size of the equation. Practically, for a sparse multi-diagonal matrix, there will only be a few non-zero diagonal vectors. We can therefore represent the complete linear equations in terms of several vectors, including the right-hand vector ${\varvec{f}}$, which will naturally be the input of the DNN model.

With this approach, we achieve the input of any multi-diagonal equation into the DNN model; thus, the DNN model can solve the linear equations of an arbitrary shape. However, in this paper, to simplify the description, we have chosen the tridiagonal equations as an example to illustrate the mechanism of the algorithm.

An arbitrary multi-diagonal matrix can have all its main diagonal elements transformed to 1 by a linear transformation, as shown by

3 $$\begin{array}{c}\left({\mathrm{diag}}_{0}\left({d}_{1},{d}_{2},\cdots ,{d}_{n}\right)+{\mathrm{diag}}_{1}\left({r}_{1},{r}_{2},\cdots ,{r}_{n-1}\right)+{\mathrm{diag}}_{-1}\left({l}_{1},{l}_{2},\cdots ,{l}_{n}\right)\right){\varvec{x}}={\varvec{f}}\\ \Rightarrow \left({I}_{n}+{\mathrm{diag}}_{1}\left(\frac{{r}_{1}}{{d}_{2}},\frac{{r}_{2}}{{d}_{3}},\cdots ,\frac{{r}_{n-1}}{{d}_{n}}\right)+{\mathrm{diag}}_{-1}\left(\frac{{l}_{1}}{{d}_{1}},\frac{{l}_{2}}{{d}_{2}},\cdots ,\frac{{l}_{n-1}}{{d}_{n-1}}\right)\right){\varvec{x}}={\left(\frac{{f}_{1}}{{d}_{1}},\frac{{f}_{2}}{{d}_{2}},\cdots ,\frac{{f}_{n}}{{f}_{n}}\right)}^{\mathrm{T}},\end{array}$$

where the original equation is represented by three diagonal vectors ${\varvec{d}}$, ${\varvec{r}}$, ${\varvec{l}}$ and the right-hand vector ${\varvec{f}}$. After the transformation shown in (3), the input vectors are reduced by one, and more significantly, the three input vectors are normalized.

The DNN model can so far be written in the form of $\mathcal{N}\left({\varvec{r}},{\varvec{l}},{\varvec{f}};\Theta \right)$, where $\Theta$ is the set consisting of the parameters and hyperparameters of the DNN model. On the other hand, the DNN model can be considered as a regression of the operator ${\varvec{y}}=\mathcal{S}\left(A,{\varvec{f}}\right)={A}^{-1}{\varvec{f}}$. We accordingly selected mean squared error (MSE) as the loss function $L\left(\cdot \right)$, shown by

4 $$L\left(\cdot \right)=\frac{1}{M}{\sum }_{i=1}^{M}{\left(\mathcal{N}\left({{\varvec{r}}}_{i},{{\varvec{l}}}_{i},{{\varvec{f}}}_{i};\Theta \right)-\mathcal{S}\left({A}_{i},{{\varvec{f}}}_{i}\right)\right)}^{2},$$

where $M$ is the size of the training set.

The training set we utilize consists of the random vectors of the Gaussian distribution

5 $$\begin{array}{c}{T}_{N}\left(\rho \right)=\left\{\left\{{{\varvec{r}}}_{i},{{\varvec{l}}}_{i},{{\varvec{f}}}_{i},{{\varvec{y}}}_{i}\right\}:{{\varvec{r}}}_{i}\in {\mathbb{R}}^{N-1};{{\varvec{l}}}_{i}\in {\mathbb{R}}^{N-1};{{\varvec{f}}}_{i}\in {\mathbb{R}}^{N};\right.\\ \left.{{\varvec{y}}}_{i}={\left(I+{\mathrm{diag}}_{1}\left({{\varvec{r}}}_{i}\right)+{\mathrm{diag}}_{-1}\left({{\varvec{l}}}_{i}\right)\right)}^{-1}{{\varvec{f}}}_{i};{r}_{ij},{l}_{ij},{f}_{ij}\sim N\left(0,{\rho }^{2}\right),j\le N\right\}\end{array}$$

where $\rho$ is the standard deviation of the training set and is treated as a given parameter in establishing the training set. It is worth noting that, although the training process of the DNN model is time-consuming, it is done offline^10^ and only once for equations of the same size.

### The residual network architecture

There are kinds of particular problems of classic deep networks^28,30,31^ that directly constrain the precision of the predicted solution, e.g., vanishing gradient, exploding gradient, and network degradation^32–35^. Focusing the above problems, especially the network degradation, Res-Net establish a direct connection between the shallow layer and deep layer imitating a short circuit. Res-Net is thus capable to fit the identity operator and forces the network to approximate the "residue" of the input–output map effectively^30,36^.

To prevent the vanishing gradient, we employ ReLU (rectified linear unit) as the activation function in the hidden layers. We denote the weight and bias of a single hidden layer as $w$ and ${\varvec{b}}$, the residual block $\mathcal{L}\left(\cdot \right)$ can be represented as

6 $$\left\{\begin{array}{l}F\left({\varvec{x}}\right)=ReLU\left(w{\varvec{x}}+{\varvec{b}}\right),\\ L\left({\varvec{x}}\right)=F\left({\varvec{x}}\right)+x.\end{array}\right.$$

The DNN model $\mathcal{N}$ can be denoted by a composite mapping, which forms

7 $$\mathcal{N}\left({\varvec{r}},{\varvec{l}},{\varvec{f}};\Theta \right)={\mathcal{L}}_{N}\circ {\mathcal{L}}_{N-1}\circ \cdots \circ {\mathcal{L}}_{1},$$

where $\circ$ stands for operator composition.

**Figure 1:**
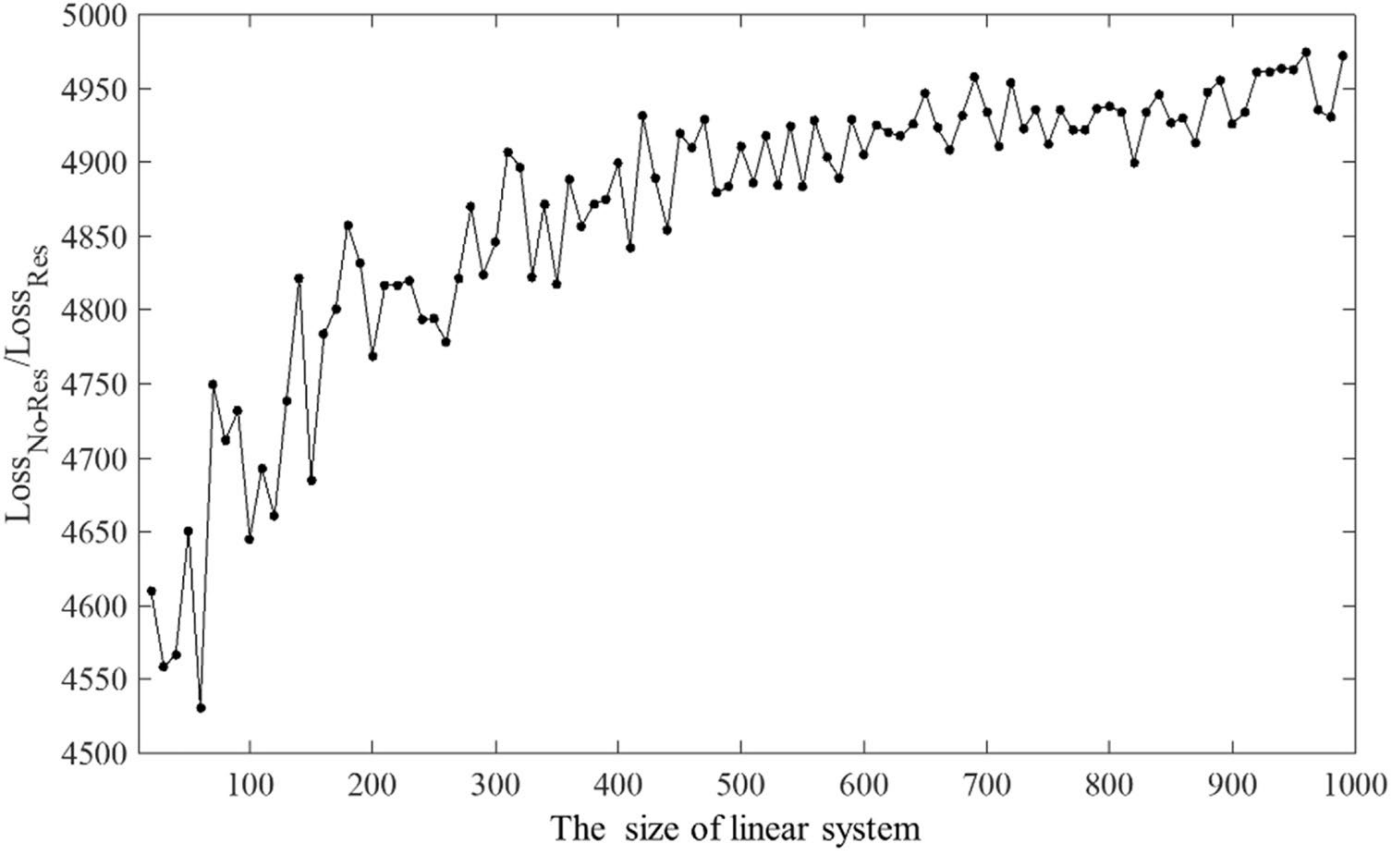
The ratio of the training loss of Res-Net and non-Res-Net at different sizes.

In addition to the Res-block, we utilize another short connection covering the whole network model. Based on this short connection, the network can be comprehended as the approximation of $\left(I-{A}^{-1}\right){\varvec{f}}$, that the output vector has smaller values and a zero mean. To evaluate the practical optimization via Res-Net, the training loss comparison between the DNN models with and without Res-Net is shown in Fig. 1.

From Fig. 1, the training loss of the DNN model containing Res-Net decreases to 1/4500–1/5000 of the original model. Moreover, the optimization of Res-Net is even more significant for large-scale linear equations. Therefore, Res-Net can be considered a reliable method to improve the precision of the DNN model.

**Figure 2:**
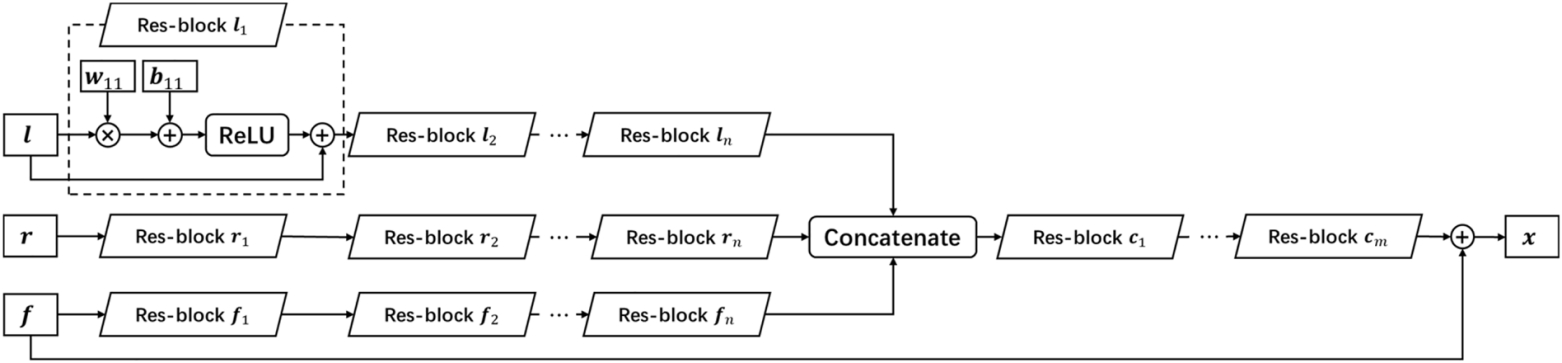
The schematic of the DNN model.

Based on the above architecture, we designed the DNN model as shown in Fig. 2. In this model, there are three input vectors whose total dimension is $\left(3N-2\right)$, while the dimension of the output vector is $N$. We thus established four groups of Res-blocks, three of which receive the input ${\varvec{r}}$, ${\varvec{l}}$, and ${\varvec{f}}$. After concatenation, the output of these three groups is fed into the left Res-block and the final output ${\varvec{x}}$ is achieved after adding ${\varvec{f}}$.

In Fig. 2, both the number of the Res-blocks (the depth of the network) and the size of the weight matrix (the number of the neurons) in each Res-block should be carefully considered based on the balance of the computational consumption and the precision. In this manuscript, we select the scheme that all the Res-block groups consist of three Res-blocks, i.e., $n=m=3$ in Fig. 2. The number of the neurons is 100 in the group ${{\varvec{l}}}_{i}$, ${{\varvec{r}}}_{i}$ and ${{\varvec{f}}}_{i}$, and 200 in the group ${{\varvec{c}}}_{i}\left(i\le 3\right)$. This scheme is a compromise choice based on the numerical tests.

### A correction iteration

Theoretically, based on the universal approximation theorem^17,18^, the network can approximate the objective function with arbitrary accuracy when the number of neurons and layers of the neural network is sufficiently large. However, an excessively large model is not acceptable for actual computation. Therefore it is impracticable to obtain an accurate solution to an arbitrary equation via an individual prediction. In addition to the Res-Net, we employed another approach to improve the numerical precision. Inspired by the classic iteration methods, e.g., CG and GMRES, we proposed a correction algorithm shown in Algorithm 1.
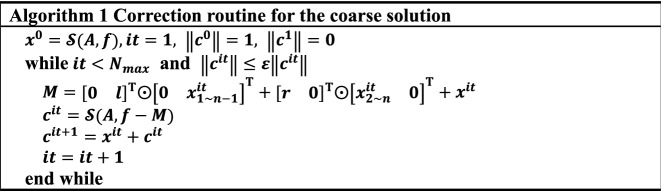


In Algorithm 1, $$\varepsilon$$ is the parameter that determines the iterative convergence criterion, which is a constant smaller than 1. The other parameter restricts the iteration number is the maximum iteration number $${N}_{max}$$. The symbol $${{\varvec{x}}}_{a\sim b}\left(a,b\in {\mathbb{N}}_{+}\right)$$ denotes the vector consisting of the $$a$$-th to $$b$$-th elements of $${\varvec{x}}$$. The operator $$\odot$$ in Algorithm 1 indicates the elementwise product of two vectors, i.e., $$\left[ {\begin{array}{*{20}c} {a_{1} } & \cdots & {a_{n} } \\ \end{array} } \right] \odot \left[ {\begin{array}{*{20}c} {b_{1} } & \cdots & {b_{n} } \\ \end{array} } \right]$$$$=\left[\begin{array}{ccc}{a}_{1}{b}_{1}& \cdots & {a}_{n}{b}_{n}\end{array}\right]$$. It can be proven that the matrix $$M$$ is the matrix–vector product $$A{{\varvec{r}}}^{it}$$.

In the calculation of matrix $M$, the tridiagonal matrix and vector production can be considered a sum of three vector operations that minimize the computational complexity and memory usage. In addition, the effect of the iterative algorithm to improve the accuracy will be evaluated by the numerical results proposed in Chapter 3.

## Precision-oriented parametric analysis

In general, the numerical precision of the proposed method could be influenced by two aspects of the input: the properties of the linear system and the parameters of the algorithm. It has been mentioned in section The representation of the linear equation that the condition number of the matrix is significantly related to the diagonal dominance. In contrast, the condition number can reflect the sensitivity of the solution. The performance of the DNN model is consequently affected by diagonal dominance. Moreover, the algorithm's relative precision (the norm ratio between the solution and error) is another essential benchmark that we evaluate in this section.

To quantify the effect of the above parameters on the precision and the convergence rate in the correction iteration, we conducted a series of numerical experiments proposed in this section. Specifically, we utilized 5000 sets of input vectors generated by standard random numbers for each set of parameters to collect the mean results. In each set of parameters, ${\rho }_{c}$ and ${\rho }_{r}$ represents the $\rho$ of the off-diagonal vector and right-hand vector, respectively.

### The influence of the input vectors

The off-diagonal vectors affect the diagonal dominance of the linear equation and the regularity of the approximated mapping. Therefore, with the increase of the off-diagonal vectors' norm, the DNN model's accuracy gradually deteriorates. The network would become invalid after reaching an uncertain threshold.

**Figure 3:**
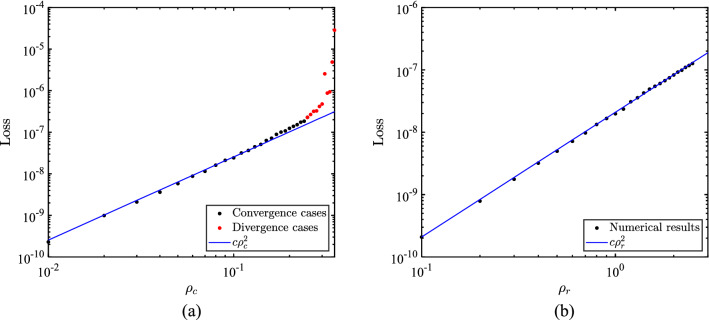
The influence of the off-diagonal elements on the training loss (Left: the influence of ${\rho }_{c}$ when ${\rho }_{r}=0.1$; Right: the influence of ${\rho }_{r}$ when ${\rho }_{c}=0.1$).

On the other hand, for a fixed matrix $A$, the mapping between the right-hand vector and solution is linear. Consequently, the norm of the solution is approximately proportional to the norm of the right-hand vector. A well-trained network should achieve high accuracy and simulate this linearity. Thus, the norm of the error would be approximately proportional to the norm of the right-hand vector. Figure 3 demonstrates the effect of the off-diagonal and the right-hand vectors on the network, respectively.

In Fig. 3, training loss represents the final value of the loss function in the training process. To investigate the effect of the single parameter, ${\rho }_{r}$ in Fig. 3a is set to 0.1 in, and ${\rho }_{c}$ in Fig. 3b is set to 0.01. In Fig. 3a, the training loss is significantly related to ${\rho }_{c}$, and the training process shows the phenomenon of non-convergence as ${\rho }_{c}$ increases to more than 0.3. Therefore, to achieve a reliable predicted solution, diagonal dominance is one of the essential factors that need to be considered.

**Figure 4:**
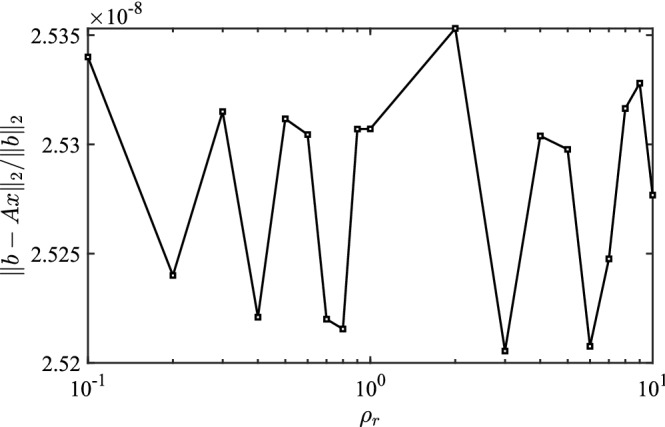
The relative error for different ${\rho }_{r}$ (${\rho }_{c}=0.01$).

According to the results shown in Fig. 3b, DNN can be considered as a regression with an approximately constant relative accuracy, i.e., the ratio $\Vert {y}^{*}-y\Vert /\Vert y\Vert$ is nearly a constant with a given network. In the view of the relative accuracy, Fig. 4 presents the relative accuracy of the network.

In Fig. 4, the relative error calculated from $\Vert {y}^{*}-y\Vert /\Vert y\Vert$ is approximately 2.53 × 10^–8^ and remains within a small range for different ${\rho }_{r}$. This demonstrates that the algorithm has an advantage in terms of numerical accuracy and is sufficient for the numerical solution of the PDE.

### The amplification factor

Since the operator $\mathcal{S}\left(A,{\varvec{f}}\right)={A}^{-1}{\varvec{f}}$ is linear to ${\varvec{f}}$, the following equality must be valid for arbitrary constant $\alpha$:

8 $$\frac{1}{\alpha }\mathcal{S}\left(A,\alpha {\varvec{f}}\right)=\mathcal{S}\left(A,{\varvec{f}}\right)$$

**Figure 5:**
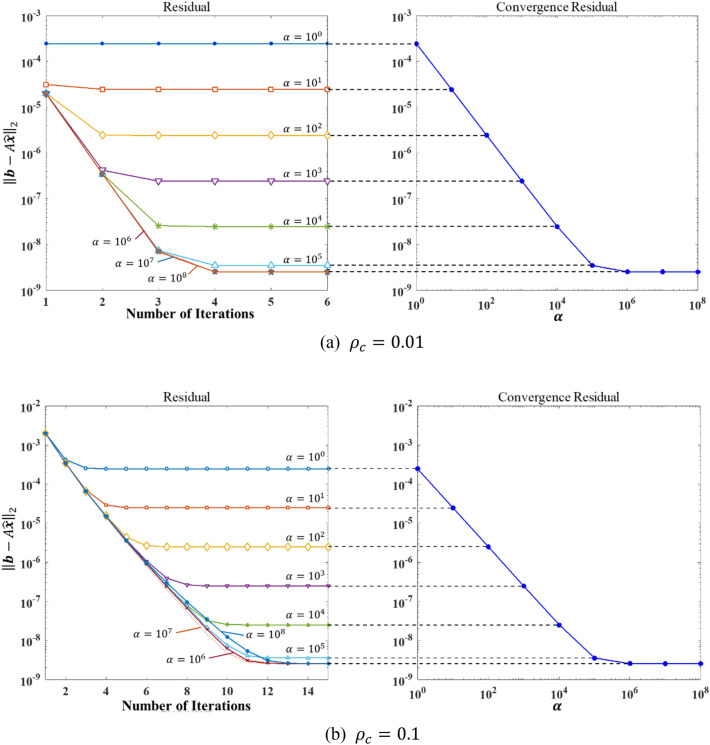
The norm of the residual vs. different $\mathrm{\alpha }$ (Left: the decreasing of the residual in each iteration; Right: the residuals with different $\alpha$).

As a regression of $\mathcal{S}\left(A,{\varvec{f}}\right)$, the output of DNN must satisfy this property as well. Nevertheless, the DNN, as a nonlinear mapping, is incapable of this linear property but approximately meets it. We were inspired by this property to conceive a factor $\alpha$, called the amplification factor, as a hyperparameter to improve the precision of the DNN model. In particular, the prediction of DNN $\mathcal{S}\left(A,{\varvec{f}}\right)$ is replaced by $\mathcal{S}\left(A,\alpha {\varvec{f}}\right)/\alpha$. According to the numerical results in section "The influence of the input vectors", the constant α can be comprehended as an amplification factor of the prediction error and drive the correction iteration to correct it easier. The comparison among the iteration processes with different $\alpha$ is shown in Fig. 5.

**Figure 6:**
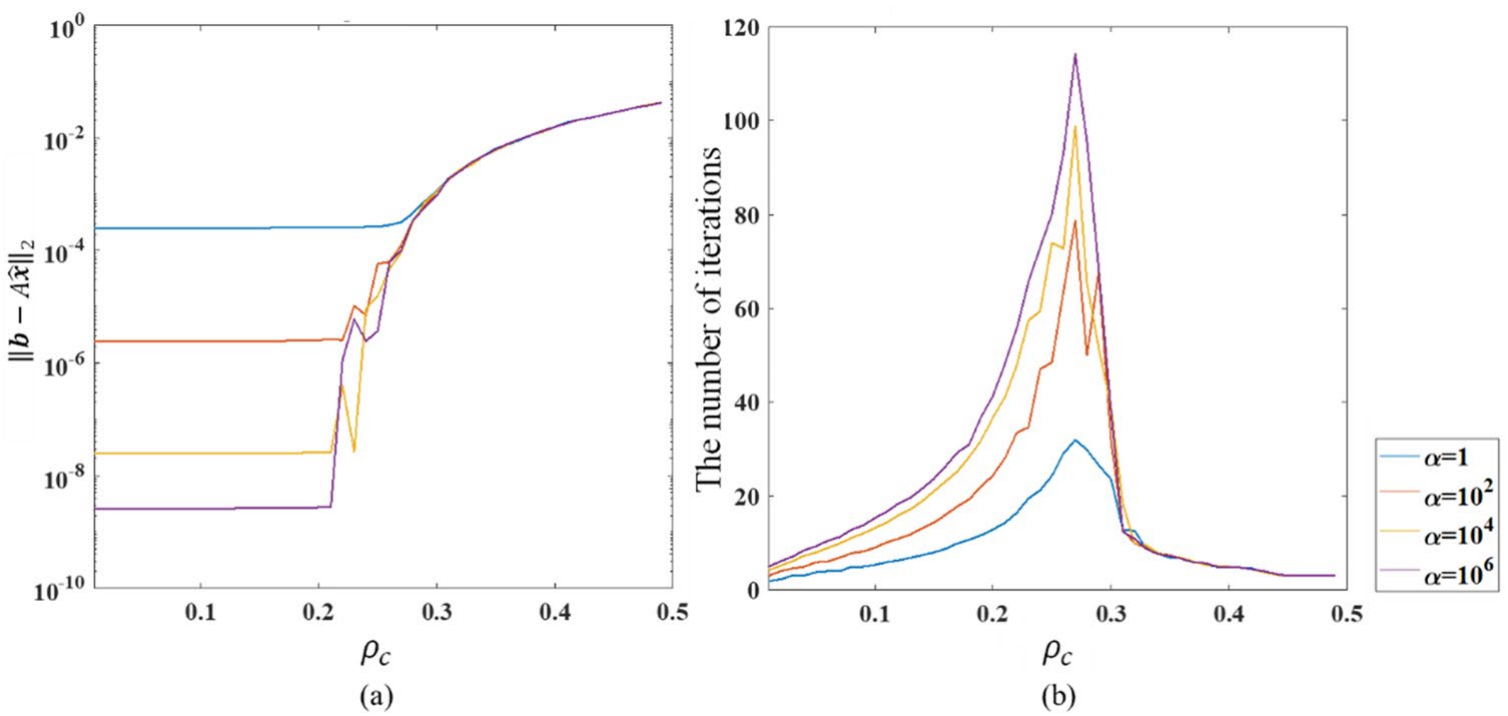
The effects of ${\rho }_{c}$ on convergence error (**a**) and the number of iterations (**b**).

In Fig. 5, as the amplification factor $\alpha$ gradually increases, the error in iterative convergence decreases significantly. However, the increase of $\alpha$ beyond a certain threshold has approximately no effect on the precision. Besides, for the given set of input vectors, $\alpha$ has little impact on the speed of convergence; thus, larger $\alpha$ also causes more iterative steps. To further demonstrate the effect of the hyperparameter $\alpha$, Fig. 6 shows the residual norm and the number of iterations with different $\alpha$.

**Figure 7:**
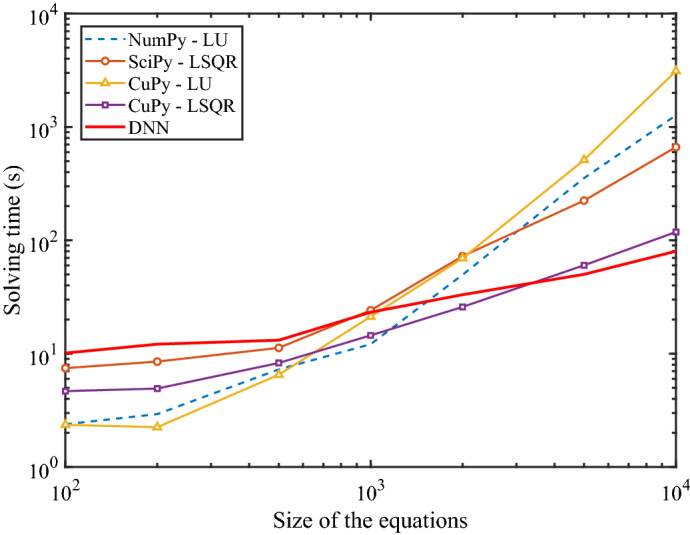
Comparison of the computation time among different solvers.

As can be observed in Fig. 6a, the influence of the off-diagonal elements on the precision has been approximately eliminated when the off-diagonal elements are smaller than the threshold (${\rho }_{c}<0.2$). Combining the results in Fig. 3, we can assert that for diagonal-dominated equations, the correction iteration practically eliminates the effect of diagonal dominance on precision. Nevertheless, for non-diagonal dominant equations (${\rho }_{c}>0.3$), the correction iteration inevitably fails to converge due to the large condition number. The amplification factor does not produce any effect in this case. Figure 7b shows that the number of iterations increases as the amplification factor increases until the iterations fail to achieve convergence. The results further demonstrate that the amplification factor practically amplifies the error of the DNN prediction and improves the numerical precision while causing an increase in the number of iterations. The number of iterations for the non-diagonal dominant equations is small due to the iterative divergence.

## Numerical experiments and validation

### Computational efficiency

To demonstrate the high efficiency of the proposed method, we compare it against the LU method in this section, as shown in Fig. 7.

The current version of our solver is developed based on the TensorFlow platform^37^, while the classic solvers in this comparison are provided by NumPy^38^ library, SciPy^39^ library (for CPU implementation), and CuPy library (for GPU implementation). On the homogeneous (CPU) platform, both the NumPy-based solver and SciPy solver natively support parallel computation. Since GPU devices can accelerate the TensorFlow platform, we utilized the CuPy-based solvers as the classic solvers on the GPU platform for a fair comparison. In this test, the NumPy-based solver is based on the LU algorithm, the SciPy-based solver utilizes the Least Square QR-factorization (LSQR) algorithm, and both of the algorithms are implemented by the CuPy-based solver.

The hardware platform in this test is Intel Xeon-W3175X CPU (28 cores, 3.1 GHz) and Nvidia RTX2080Ti GPU. In every single test, we solved 5000 random linear equations of a given size and calculated the average solving time.

In Fig. 7, as the size of the matrix increases, the computation time of the proposed DNN-based algorithm grows much slower than other classic solvers, and for matrices of size over 1000, this algorithm can achieve an acceleration of 10 to 100 times compared with the CPU-implemented algorithms.

The CuPy-based LU algorithm performs best in small equations but worst in large ones due to the bandwidth constraint between the CPU and GPU. The CuPy-based LSQR algorithm keeps most of the computation subroutines on the GPU, thus having advantages over the CPU-implemented algorithms. However, as the matrix increases, its time increases faster than the DNN-based solver; therefore, the DNN-based algorithm is more suitable for solving large-scale problems.

### The heat conduction equation

In order to further highlight the high efficiency and accuracy of our algorithm, a set of PDEs is solved numerically using the proposed method. The first case is a 2D heat-conduction equation with mixed boundary conditions on a rectangle area shown by

9 $$\left\{\begin{array}{l}{\partial }_{t}u-\beta \left({\partial }_{xx}^{2}u+{\partial }_{yy}^{2}u\right)=0, \left(x,y\right)\in \Omega \\ {\frac{\partial u}{\partial n}\left.\right|}_{y=\mathrm{0,1}}=0\\ {u\left.\right|}_{x=0}=0, {u\left.\right|}_{x=1}=1\end{array}\right.$$

where the problem domain $\Omega =\left\{\left(x,y\right)|0\le x\le 1, 0\le y\le 1\right\}$,$\beta$ is the conduction coefficient, assumed as a constant in this problem.

In the present case, the parameters of the simulation are shown in Table 1.

**Table 1:** Parameters of the 2D heat-conduction equation.

Parameter	Value
$\beta$	0.1
$\Delta t$	0.001
$\Delta x$	0.0025
$\Delta y$	0.0025
$T$	1.0

**Figure 8:**
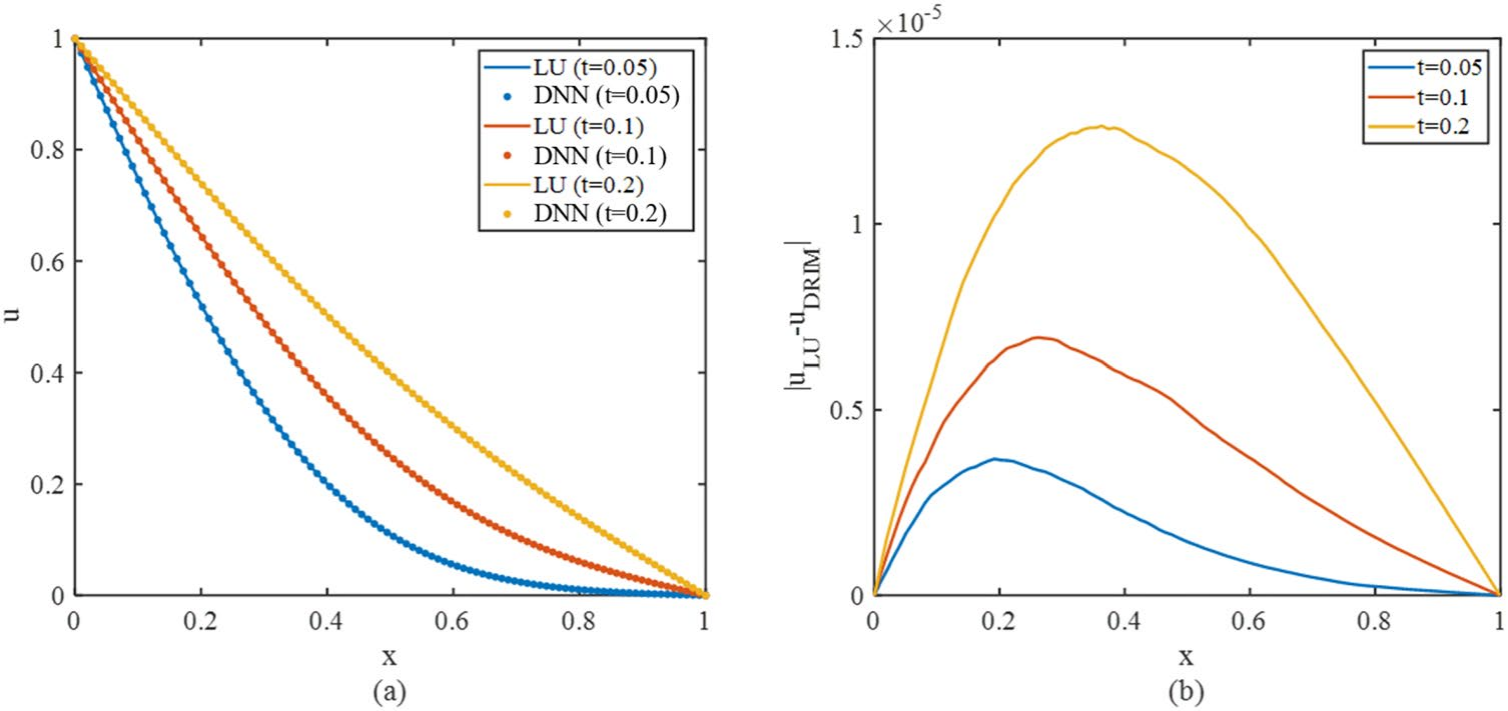
The numerical solution to the heat conduction equation at y = 0.5 (**a**) and the error distribution (**b**).

The ADI scheme with 2nd order spatial precision and 1st temporal precision is utilized, which forms

10 $$\left\{\begin{array}{c}{u}_{i,j}^{n+\frac{1}{2}}-\frac{1}{{\tau }_{x}^{+}\Delta {x}^{2}}{u}_{i+1,j}^{n+\frac{1}{2}}-\frac{1}{{\tau }_{x}^{+}\Delta {x}^{2}}{u}_{i-1,j}^{n+\frac{1}{2}}=\frac{1}{{\tau }_{x}^{+}{\tau }_{y}^{-}}{u}_{i,j}^{n}+\frac{\beta }{{\tau }_{x}^{+}\Delta {y}^{2}}\left({u}_{i,j+1}^{n}+{u}_{i,j-1}^{n}\right)\\ {u}_{i,j}^{n+1}-\frac{1}{{\tau }_{y}^{+}\Delta {y}^{2}}{u}_{i,j+1}^{n+1}-\frac{1}{{\tau }_{y}^{+}\Delta {y}^{2}}{u}_{i,j-1}^{n+1}=\frac{1}{{\tau }_{y}^{+}{\tau }_{x}^{-}}{u}_{i,j}^{n+\frac{1}{2}}+\frac{\beta }{{\tau }_{y}^{+}\Delta {x}^{2}}\left({u}_{i+1,j}^{n+\frac{1}{2}}+{u}_{i-1,j}^{n+\frac{1}{2}}\right)\end{array}\right.$$

where ${\tau }_{x}^{\pm }=2{\left(\Delta {t}^{-1}\pm\Delta {x}^{-2}\right)}^{-1}$. The tridiagonal linear equation can represent each semi-step, and the proposed method can thus solve the linear equation. The comparison between the numerical solutions at the first 200 timesteps is shown in Fig. 8.

To demonstrate the error distribution across the field more clearly, we have chosen the x-u section to illustrate the error distribution in Fig. 8. Although the LU linear solver achieves the reference solution, all the classic solvers mentioned in Fig. 7 can achieve an approximately precise solution as well. The error across the area is approximately ${10}^{-6}$, in the same order of magnitude as the discretization error of the difference scheme in Eq. (8).

### The convection–diffusion equation

This section presents a nonlinear problem solved by the proposed method. The Burgers equation is one of the fundamental PDEs in various fields such as nonlinear acoustics, gas dynamics, fluid mechanics, etc.^40^. The Burgers equation was first introduced by H. Bateman^41^ and later studied by J. M. Burgers^42^ in the theory of turbulence. Considering the nonlinear Burgers equation shown by

11 $$\left\{\begin{array}{l}{\partial }_{t}u-u{\partial }_{x}u-\beta {\partial }_{xx}^{2}u=0, x\in \left[\mathrm{0,1}\right]\\ {u\left.\right|}_{x=\mathrm{0,1}}=0,\\ u{\left.\right|}_{t=0}=\mathrm{sin}2\pi x,\end{array}\right.$$

where $\beta$ is the factor of diffusion.

The difference scheme with 2nd precision we selected is shown by

12 $$\begin{array}{c}\left(1-\alpha \left({u}_{i+1}^{n}-{u}_{i-1}^{n}\right)+2\widehat{\beta }\right){u}_{i}^{n+1}+\left(\alpha {u}_{i}^{n}-\widehat{\beta }\right){u}_{i-1}^{n+1}-\left(\alpha {u}_{i}^{n}+\widehat{\beta }\right){u}_{i+1}^{n+1}\\ ={u}_{i}^{n}+\widehat{\beta }\left({u}_{i+1}^{n}-2{u}_{i}^{n}+{u}_{i-1}^{n}\right),\end{array}$$

where the constant $\alpha =1/4\Delta x$, $\widehat{\beta }=\beta \Delta t/2\Delta {x}^{2}$ the parameter is set as time step $\Delta t=0.001$, spatial step $\Delta x=1/400$, $\beta =0.25$.

**Figure 9:**
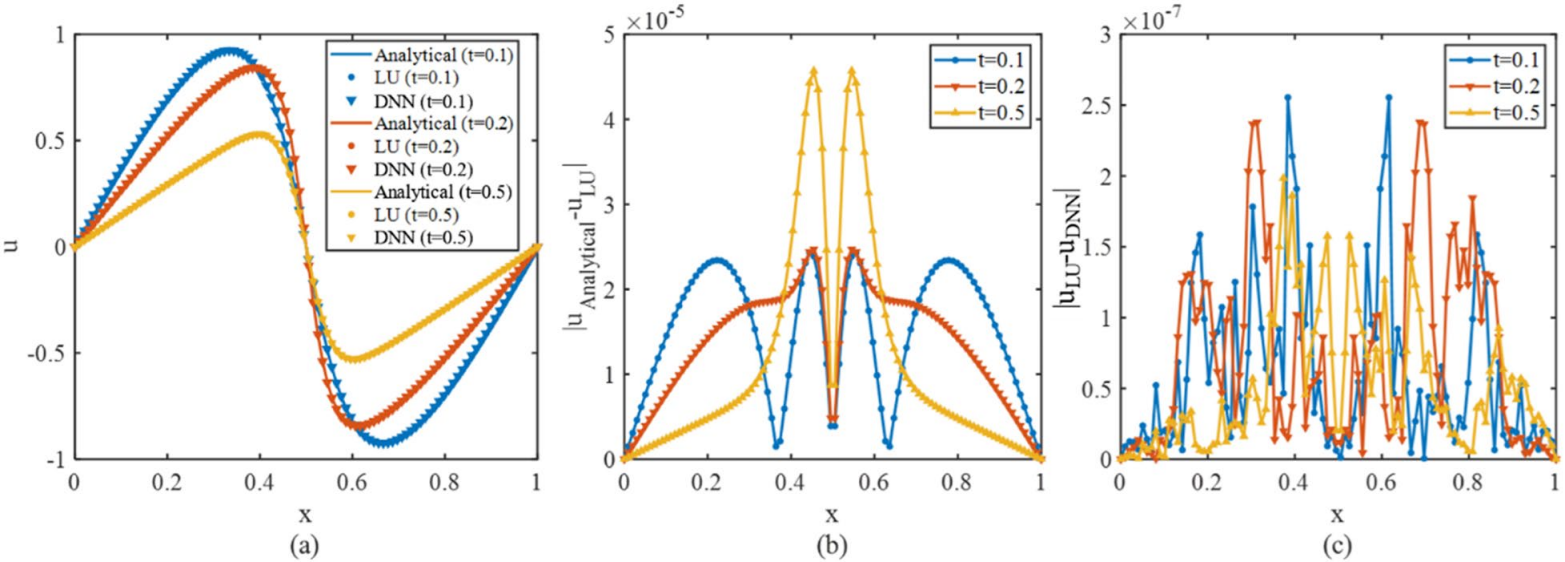
The numerical and the analytical solution to the convection–diffusion equation (**a**) and the error distribution (**b**,**c**).

Analytic solutions exist for this equation; thus, the numerical results can be employed to evaluate the precision of both the differential format and the proposed algorithm. Figure 9 shows the numerical results and error distribution in the first 500 timesteps.

In Fig. 9, the error of both two algorithms across the entire field is approximately ${10}^{-7}$, as shown in Fig. 9c, while the discretization error of the difference scheme is approximately ${10}^{-5}$, as shown in Fig. 9b.

Both linear and nonlinear equation examples illustrate the high precision of the proposed method, while most other NN-based solvers, including PINN^20^, hp-VPINN^21^, etc.^26,43^, have errors in the magnitudes range of ${10}^{-2}$ to ${10}^{-3}$.

## Conclusion

This paper introduces a DNN-based algorithm to solve the linear equation with high efficiency and precision. The algorithm combines the Res-Net architecture and the correction iteration method.

1. Based on the DNN model, the proposed method has high computation efficiency and the native hardware compatibility on GPU and other hybrid platforms. Compared with classic methods, this method can simultaneously solve numerous linear equations with a low computation complexity.
2. We employed the Res-Net architecture to avoid the network degradation of the DNN and consequently improve the precision of the DNN model. The proposed method also includes the correction iteration to reduce the error further to achieve acceptable algorithm precision iteratively.
3. We verify the proposed method's reliability, numerical precision, and computational efficiency by applying it to solve practical PDEs (including linear and nonlinear equations) in this paper. In addition, we provide several numerical results to evaluate the numerical precision of the proposed method and its affecting factors, e.g., the diagonal dominance of the input matrix.
4. Like the other algorithms based on the DNN, the performance of the proposed method is also significantly affected by the factors of input variables. We investigate some typical elements, e.g., the diagonal dominance and the norm of the right-hand vector, based on the numerical results.
5. Inspired by the properties of the analytical solution of the linear equation, we conceive the amplification factor to improve the precision of the proposed method. The amplification factor can efficiently reduce the error of the solution and eliminate the effect of the diagonal dominance but causes more iteration steps.
6. According to the numerical results, including a nonlinear problem and linear problem, the proposed method is reliable in solving the PDEs numerically with high computational efficiency and sufficient precision.

For a long time, accuracy and interpretability have been fatal problems in exploring the DNN for applications in numerical computation. The method proposed in this research provides an innovative idea with a reference value. However, the currently proposed method still has some limitations, including the inability to solve non-diagonally dominated equations.

Some limitations still exist in the current version of the algorithm, such as difficulties with rigid problems. In our subsequent research, we will improve the proposed algorithm to address these limitations and continue to investigate the combination of the proposed DNN models with classic algorithms, e.g., the preconditioner based on the proposed method. Moreover, the proposed method has been proven effective in accelerating the solving process of multi-diagonal equations. We will continually explore its application to three-dimensional problems and other PDEs.

## Supplementary Information

Supplementary Information.

## Data Availability

The training data set in the manuscript is composed of random numbers (the generating method is introduced in section "Methodology" of the manuscript). The trained model we obtained will be submit as attachment (h5 file), which is also the data file needed to achieve the results of this manuscript. The other data used or analysed during the current study available from the corresponding author on reasonable request.
